# Supplementary material for: Comparative study of two Rift Valley fever virus field strains originating from Mauritania
Source: PLoS Negl Trop Dis. 2024 Dec 9;18(12):e0012728. doi: 10.1371/journal.pntd.0012728 (PMC11658707; doi:10.1371/journal.pntd.0012728)
Supplement: S1 Table — (PDF) [file pntd.0012728.s002.pdf]

S1 Table: Mapping statistics of the high throughput sequencing data.

Strain	Total reads	L segment		M segment		S segment	
		Mapped reads	Average depth	Mapped reads	Average depth	Mapped reads	Average depth
MRU25010-30	2067461	224815 (10.87%)	9332	42895 (2.07%)	3062	53173 (2.57%)	8509
MRU2687-3	1598621	125040 (7.82%)	5160	151253 (9.46%)	10448	63700 (3.98%)	9926
ZH548	905582	35163 (3.88%)	1353	39421 (4.35%)	2614	8063 (0.89%)	1199
