# Supplementary material for: Comparative study of two Rift Valley fever virus field strains originating from Mauritania
Source: PLoS Negl Trop Dis. 2024 Dec 9;18(12):e0012728. doi: 10.1371/journal.pntd.0012728 (PMC11658707; doi:10.1371/journal.pntd.0012728)
Supplement: S2 Table — Amino acid substitutions are classified by viral segment and related protein. Their position was determined relative to the known start codon. Note that the numbering of M segment proteins (NSm, Gn, and Gc) starts from AUG1 used to translate p78. Amino acid residues can be classified in four groups based on their polarity (non-polar, polar with no charge on R group, polar with negative charge on R group, and polar with positive charge on R group). Amino acid residues conserved between MRU25010-30 and MRU2687-3 are uncoloured. Non-conserved amino acid residues between these two strains but identical between MRU25010-30 and ZH548 strains are coloured in light grey. Non-conserved amino acid residues between MRU25010-30 and MRU2687-3, and from the same group are coloured in grey. Non-conserved amino acid residues and from a different group are coloured in dark grey. (PDF) [file pntd.0012728.s003.pdf]

S2 Table: Amino acid substitutions observed between the consensus sequences of MRU25010-30, MRU2687-3, and ZH548 strains.

Segment	Protein	Position	MRU25010-30	MRU2687-3	ZH548
S	NSs	23	I	I	F
		90	V	I	I
		111	V	I	I
		167	V	V	A
		217	A	A	V
		242	V	V	I
		245	V	I	I
		262	V	A	V
	N	159	E	E	G
M	NSm	42	R	G	G
		126	V	I	I
	Gn	232	Q	Q	L
		269	V	I	V
		360	D	N	D
		384	K	T	T
		492	I	V	V
		566	G	G	D
		595	V	I	I
		605	K	R	R
		615	K	R	R
		631	V	V	I
		659	A	V	V
	Gc	739	D	E	E
		747	I	I	L
		1059	T	T	S
L	L	23	Y	Y	F
		120	M	T	M
		157	D	G	G
		177	E	D	E

		249	K	R	R
		278	N	N	S
		288	V	A	A
		302	I	V	V
		336	I	V	V
		350	K	R	R
		406	V	M	M
		407	D	G	G
		411	G	S	S
		470	N	N	S
		493	R	K	R
		663	T	T	A
		840	I	V	I
		1333	I	V	V
		1698	I	V	V
		1751	M	I	I
		1760	V	I	V
		1852	H	Y	H
		1926	R	K	R
		1960	E	D	D
		1984	N	N	D
		2033	T	A	A

Amino acid substitutions are classified by viral segment and related protein. Their position was determined relative to the known start codon. Note that the numbering of M segment proteins (NSm, Gn, and Gc) starts from AUG1 used to translate p78. Amino acid residues can be classified in four groups based on their polarity (non-polar, polar with no charge on R group, polar with negative charge on R group, and polar with positive charge on R group). Amino acid residues conserved between MRU25010-30 and MRU2687-3 are uncoloured. Non-conserved amino acid residues between these two strains but identical between MRU25010-30 and ZH548 strains are coloured in light grey. Non-conserved amino acid residues between MRU25010-30 and MRU2687-3, and from the same group are coloured in grey. Non-conserved amino acid residues and from a different group are coloured in dark grey.
