# Supplementary material for: Comparative study of two Rift Valley fever virus field strains originating from Mauritania
Source: PLoS Negl Trop Dis. 2024 Dec 9;18(12):e0012728. doi: 10.1371/journal.pntd.0012728 (PMC11658707; doi:10.1371/journal.pntd.0012728)
Supplement: S3 Fig — Coverage (1) and Shannon entropy (2) for S segments of MRU2687-3 (A), MRU25010-30 (B), and ZH548 (C). (PDF) [file pntd.0012728.s006.pdf]

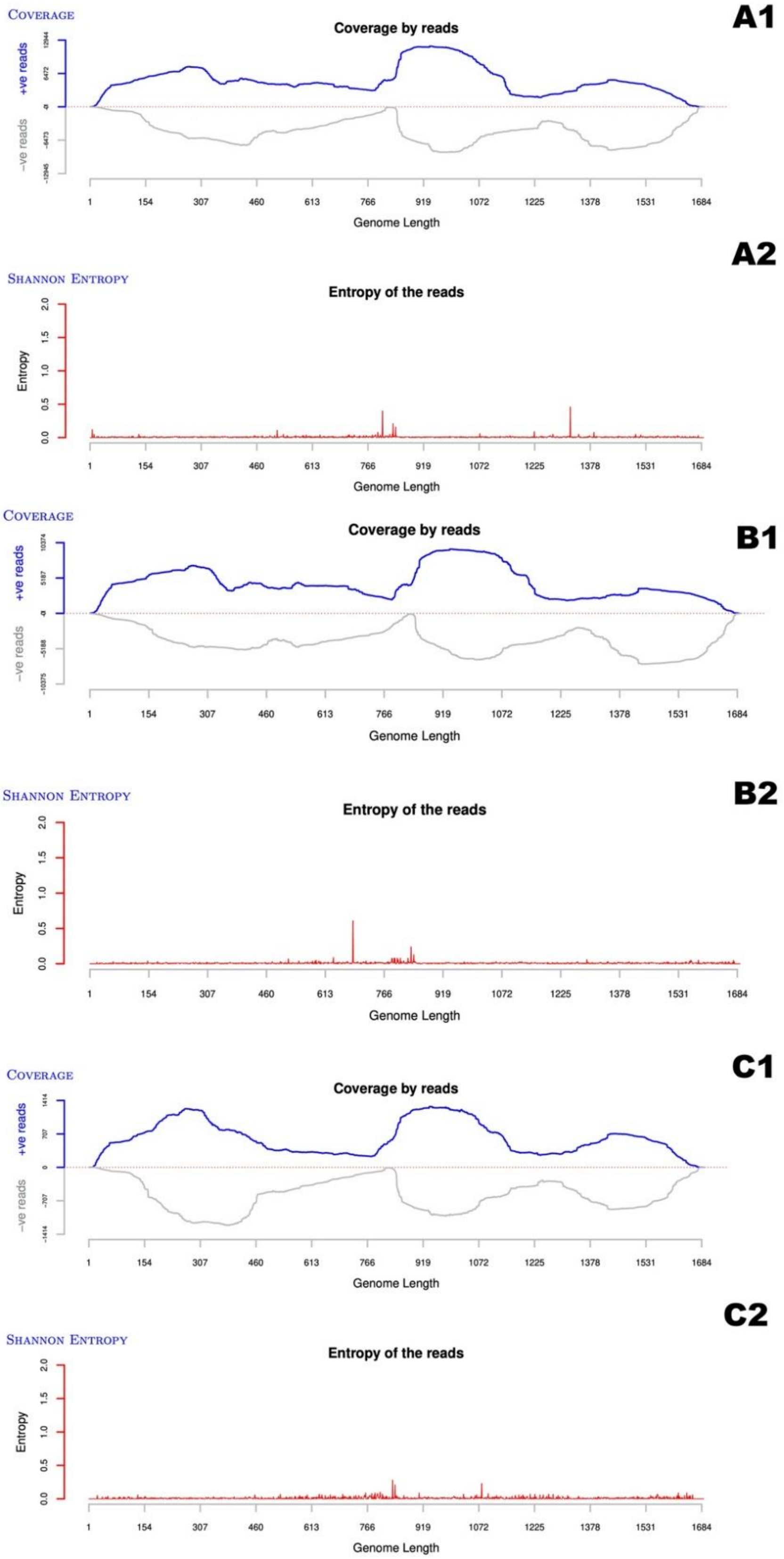

S3 Fig: Coverage and Shannon entropy plots for S segments. Coverage (1) and Shannon entropy (2) for S segments of MRU2687-3 (A), MRU25010-30 (B), and ZH548 (C).
