# Supplementary material for: Comparative study of two Rift Valley fever virus field strains originating from Mauritania
Source: PLoS Negl Trop Dis. 2024 Dec 9;18(12):e0012728. doi: 10.1371/journal.pntd.0012728 (PMC11658707; doi:10.1371/journal.pntd.0012728)
Supplement: S4 Fig — Nucleotides at position 10 is either an uracile (U) or a cytosine (C) in MRU2687-3 and ZH548 strains. (PDF) [file pntd.0012728.s007.pdf]

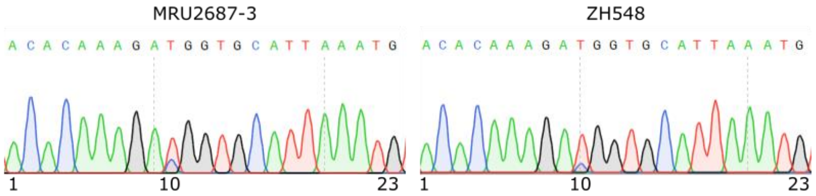

S4 Fig : DNA sequencing chromatograms of RACE-PCR products amplified from 5'UTR of anti-genomic M segments. Nucleotides at position 10 is either an uracile (U) or a cytosine (C) in MRU2687-3 and ZH548 strains.
