# Supplementary material for: Comparative study of two Rift Valley fever virus field strains originating from Mauritania
Source: PLoS Negl Trop Dis. 2024 Dec 9;18(12):e0012728. doi: 10.1371/journal.pntd.0012728 (PMC11658707; doi:10.1371/journal.pntd.0012728)
Supplement: S5 Fig — Mice have been weighted before infection and then every day during the course of the experiment. Weight percentage was calculated relative to that at Day 0. Black dots represent the last measure recorded before the death of the animal. (PDF) [file pntd.0012728.s008.pdf]

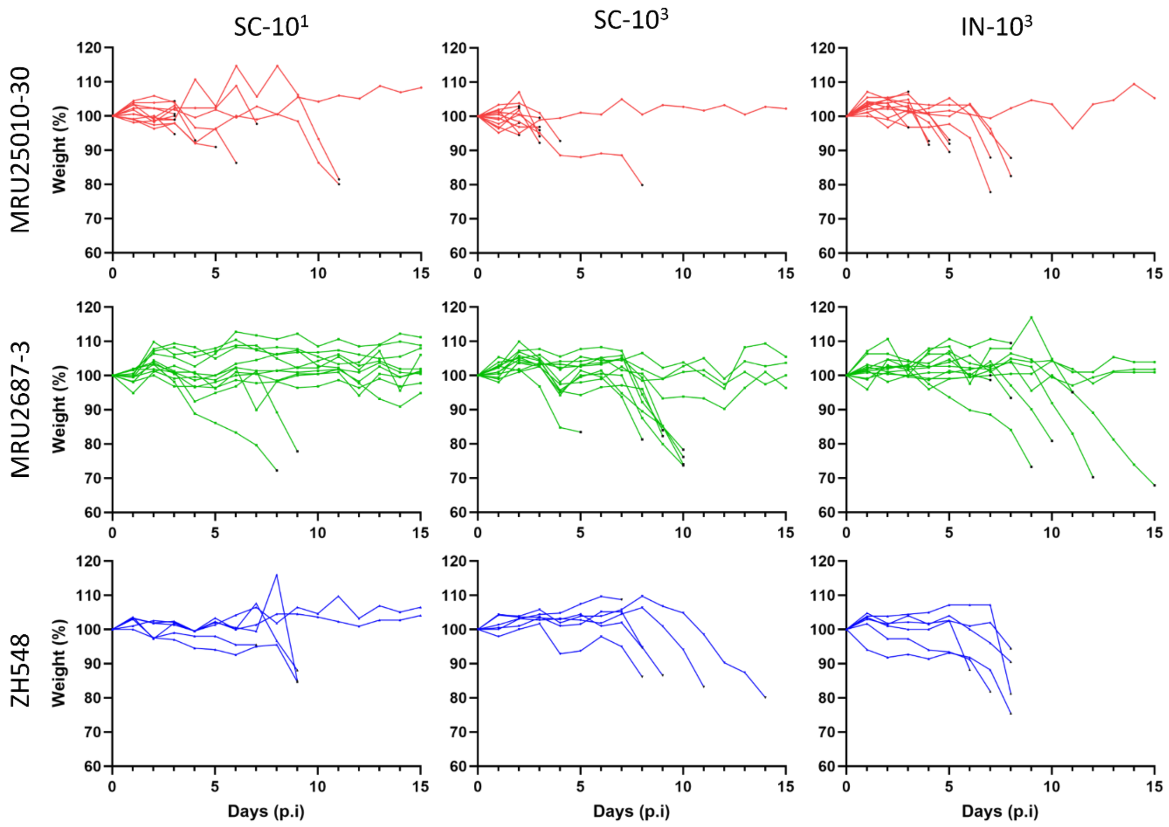

S5 Fig: Effect of RVFV infection on BALB/c mice body weight. Mice have been weighted before infection and then every day during the course of the experiment. Weight percentage was calculated relative to that at Day 0. Black dots represent the last measure recorded before the death of the animal.
