# Supplementary material for: Comparative study of two Rift Valley fever virus field strains originating from Mauritania
Source: PLoS Negl Trop Dis. 2024 Dec 9;18(12):e0012728. doi: 10.1371/journal.pntd.0012728 (PMC11658707; doi:10.1371/journal.pntd.0012728)
Supplement: S6 Fig — Anti-RVFV antibodies within mice sera were detected using in-house IgM and IgG ELISAs (Enzyme-Linked ImmunoSorbent Assay), as previously described [41,48]. Briefly, RVFV antigens were prepared from RVFV MP12 strain infected VeroE6 cells (MOI = 0,01; 2 days post-infection). For IgM detection, 96 -well plates (Nunc Maxisorp, Thermo Fisher Scientific) were coated with rabbit anti-mouse IgM antibody (100 μL/well, 1:400 dilution; Sigma, SAB3701197) and incubated with 100 μL/well of 1:100 dilution of mice sera. RVFV antigens were subsequently detected with hyperimmunised sera from hamster infected with ZH501 strain and Goat anti-Hamster IgG (H+L)-HRP (Horseradish Peroxidase) conjugated antibody. For IgG detection, plates were coated with RVFV antigens, further incubated with 100 μL/well of 1:100 dilution of mice sera and subsequently with HRP-conjugated rabbit anti-mouse IgG (whole molecule, 1:5000, Sigma, A9044). HRP enzymatic activity was revealed using TMB substrate (Thermo Fischer Scientific). Optical density at 450 nm (OD450) was measured using a TECAN microplate reader. ELISA measurement of IgM and IgG antibodies of mice infected with field strains or ZH548. The sera were collected at days 3, 4–6, 7–10, and 11–15 pi. At the indicated day, black bars represent dead mice, grey bars seroconverted mice, and white bars mice with non-detectable IgM or IgG antibodies. (PDF) [file pntd.0012728.s009.pdf]

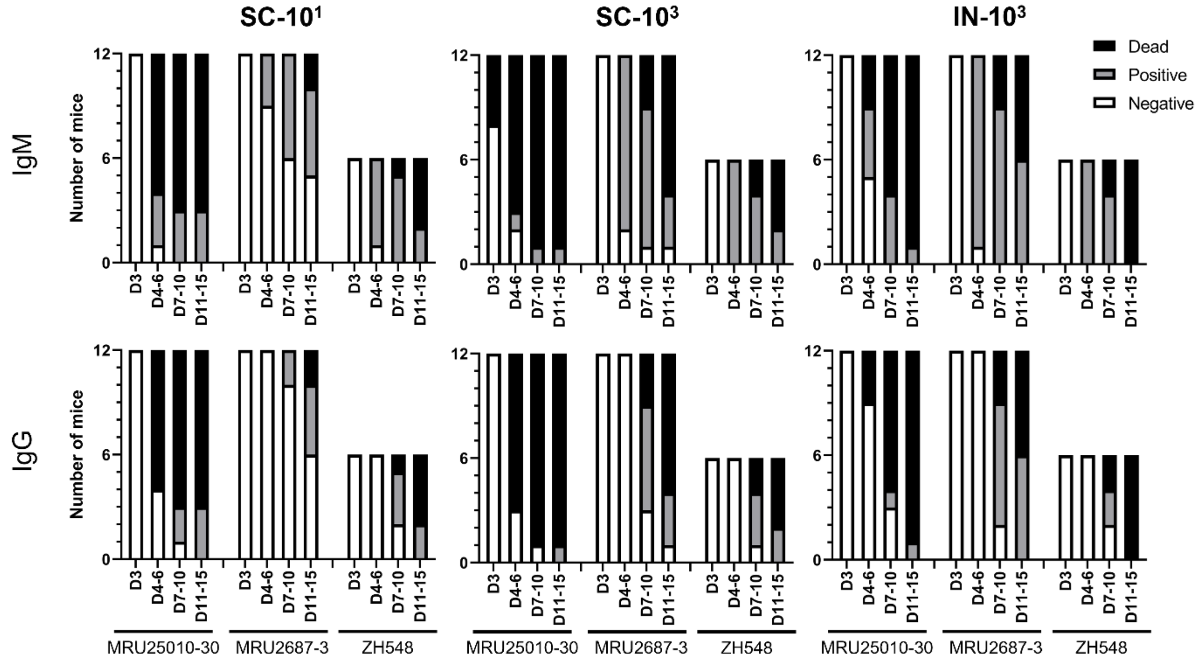


**S6 Fig: Kinetics of seroconversion of mice infected with MRU25010-30, MRU2687-3, and ZH548 strains.** Anti-RVFV antibodies within mice sera were detected using in-house IgM and IgG ELISAs (Enzyme-Linked ImmunoSorbent Assay), as previously described [41,48]. Briefly, RVFV antigens were prepared from RVFV MP12 strain infected VeroE6 cells (MOI=0,01; 2 days post-infection). For IgM detection, 96 -well plates (Nunc Maxisorp, Thermo Fisher Scientific) were coated with rabbit anti-mouse IgM antibody (100 µL/well, 1:400 dilution; Sigma, SAB3701197) and incubated with 100 µL/well of 1:100 dilution of mice sera. RVFV antigens were subsequently detected with hyperimmunised sera from hamster infected with ZH501 strain and Goat anti-Hamster IgG (H+L)-HRP (Horseradish Peroxidase) conjugated antibody. For IgG detection, plates were coated with RVFV antigens, further incubated with 100 µL/well of 1:100 dilution of mice sera and subsequently with HRP-conjugated rabbit anti-mouse IgG (whole molecule, 1:5000, Sigma, A9044). HRP enzymatic activity was revealed using TMB substrate (Thermo Fischer Scientific). Optical density at 450 nm (OD450) was measured using a TECAN microplate reader. ELISA measurement of IgM and IgG antibodies of mice infected with field strains or ZH548. The sera were collected at days 3, 4-6, 7-10, and 11-15 pi. At the indicated day, black bars represent dead mice, grey bars seroconverted mice, and white bars mice with non-detectable IgM or IgG antibodies.
