# Supplementary material for: Comparative study of two Rift Valley fever virus field strains originating from Mauritania
Source: PLoS Negl Trop Dis. 2024 Dec 9;18(12):e0012728. doi: 10.1371/journal.pntd.0012728 (PMC11658707; doi:10.1371/journal.pntd.0012728)
Supplement: Attachment — Submitted filename: Rebuttal_Letter_Chabert_et_al_2024_R1.pdf [file pntd.0012728.s012.pdf]

Brian Byrd, Guest Editor

David Safronetz, Section Editor

PLOS Neglected Tropical Diseases

28 August 2024

Dear Editors

RE: Comparative study of two Rift Valley fever virus field strains originating from Mauritania.
(Revised manuscript of PNTD-D-24-00500: Comparative study of two Rift Valley fever virus field strains circulating in Mauritania in 2010 and 2013 reveals the high virulence of the MRU25010-30 strain isolated from camel)

We would like to thank you and the reviewers for the time spent on our manuscript and for their helpful recommendations and suggestions.

Below, we provide a "point-by-point" reply. In order to facilitate the editorial process, we inserted our replies directly underneath the relevant text of the reviewers (in red), which we cut and pasted from the Editors' letter and additional reviewers' documents. In addition, as recommended, we provided two versions of the revised manuscript: one with track changes denoting where the text has been changed and a clean version uploaded as manuscript file.

Reviewer #1:

Reviewer #1 was very supportive of the original manuscript stating that the conclusions "are supported by the data obtained", "the manuscript is well written" and the results are "well presented" "both in figures and words", despite "few grammatical inaccuracies" that we believe have been corrected in the revised version.

General comments

".., the use of the words virulence (lines 2 and 53) and pathogenicity (line 79 & so on) need to be context sensitive, such that the aspects relating to the virus and those relating to the host can be clearly understood."

We agree with reviewer #1 and made the following changes:

Line 2: The title has been modified to: *"Comparative study of two Rift Valley fever virus field strains originating from Mauritania."* This change is also proposed regarding comments from Reviewer #3 (see below).

Line 53: we clarified the context by adding *"in mammalian hosts"* after virulence

Line 79: we changed "RVF pathogenicity" to *"RVFV pathogenesis"*

Additionally, we verified and modified, when necessary, the manuscript to ensure that the use

of these terms is properly contextualized (see track changes version of the manuscript).

"The authors should bear in mind also that recent field isolates of pathogens tend to be more pathogenic. This decreases with passages and/or propagation in vitro or in vivo. Although the factors which underlie this are not understood, some could confound observations such as the ones made in this study."

The main goal of this study is to compare two RVFV field strains that undergo the same *in vitro* passage history. Additionally, we fully sequenced the genome of ZH548 strain. Even if we are unable to trace the whole passage history of ZH548 strain used in this study, we found that Seg-L and Seg-S are 100% identical to the reference sequences (NC_014397 and NC_014395, respectively), when we identified only one non-synonymous mutation within Seg-M consensus sequence (nt position 1627 within negative strand; Ile to Leu in Gc) compared to the reference sequence (NC_014396). Interestingly, this position exists as a population (Table 1, ZH548, Gc, 747: Leu (88%) and Ile (12%)).
Nevertheless, we agree with the reviewer' comment and added the following sentence in the discussion to emphasis this point (lines 529-532 in the revised manuscript) in addition to two other parts of the discussion related to this topic already present in the original manuscript (lines 583-585 and lines 596-601 in the revised manuscript) : *"However, it is difficult to compare the data obtained in this study with those generated by others, as passage history (number of passages and/or cell lines used) of RVFV isolates may influence their virulence in mice."*

Specific comments

**Line**	**Comment**
34-35	isolated from, not in
54 and 124:	it is the genomes of the viruses, not the viruses, that were sequenced
83	outbreak refers to disease, in this case Rift Valley fever, not RVFV
95	change "is" to "has been".
124	insert the genomes of between the words sequenced and these
321	acids should be acid
433	replace have with had
435 & 472	replace has been with was
523	replace participates with contributes, etc

We acknowledged all the specific comments made by reviewer #1 and corrected the manuscript accordingly.

Reviewer #2:

We thank reviewer #2 for her/his useful suggestions and for being comprehensive about the difficulty for non-English speakers to write in English. The reviewer #2 states: "This study needs to confirm the presence of replicating RVFV is some of the samples", and "…, detecting viral RNA does not mean that any infectious virus was present. Therefore, determining a viremia via RT-qPCR will often give misleading information and greatly extend the actual viremic period. I did not see anywhere in the manuscript that infectious virus was detected from any of the samples. Unfortunately, I have seen several studies where pieces of viral RNA were detected and the authors claimed to have found virus, but upon checking, no infectious virus was present. Some of these sample need to be tested for infectious RVFV."

We addressed this concern of reviewer 2 by performing viral titrations in liver and brain samples collected in mice euthanized during the experiment. We modified Figure 5 to include this new set of data. More detailed answers are given in specific comments 8, 9 and 12 (please see below).

Specific comments

1. Title: Minor, but shouldn't it be "isolated from a camel?"

The title has been modified to: *"Comparative study of two Rift Valley fever virus field strains originating from Mauritania."*

2. Lines 73-80: Shouldn't you also mention that humans can be infected with RVFV by the bite of an infectious mosquito?

We modified the sentence to make that point clearer. *"Humans are usually infected by RVFV through either bites from infected mosquitoes or direct exposure to contaminated fluids, …"* (lines 73-74 in the revised manuscript)

3. Line 90: Yes, there were 63 recognized and reported human cases, but how many additional human infections/cases were there that were not recognized or reported. As stated in the current paper, it implies that the case fatality rate was 21%, which is very high for RVF in humans. The cited paper also stated, "a total of 63 cases among humans, including 13 deaths, had been officially reported, but the true number is probably much higher due to the remoteness of the affected area." I think you should include a statement that the number of cases was likely to be underreported and was actually much greater.

We modified the sentence as suggested by reviewer #2 *"among 63 human cases, although the number of cases is probably under evaluated [19]"*. (lines 90-91 in the revised manuscript)

4. Lines 229-233: What was the volume of inoculum used to infect mice either IN or SC? If you are going to repeat the methods on lines 376-378. You should also include the volumes. Did

you use the same volume for SC as for IN? If so, that was a lot to inject IN in a mouse and may have done some tissue damage resulting in some SC inoculation. If not, then did you use difference concentrations of stock virus?

We agree that it is an important point, and that it is not recommended to use the same volumes for both IN and SC groups. We modified the text to precise the experimental procedure used in this study: *"Volumes of infections were 20 µl per nostril for IN inoculation and 100 µl for SC inoculation."* (lines 246-247 in the revised manuscript). Therefore, in our experimental procedure, the viral concentrations of the inocula are different between IN and SC conditions in order to obtain the same infectious dose in both conditions.

5. Lines 379-384 (Figure 3): several comments:

a. Why write "10PFU (10) or 103PFU (103) of…" instead of "10^1 or 10^3 PFU of…?"Please note that I had to use the "^" character as I cannot use a superscript in the review.

We thank Reviewer #2 for this suggestion, and we have replaced 10 with 10^{1} throughout the manuscript and in Figures 3, 4, 5, S5 and S6.

b. "eight weeks old…" should be "8 weeks old…"

We corrected the text accordingly.

c. I assume that there were 12 mice for each dose/route for the two Mauritanian strains of RVFV. Is that correct?

This is correct. As this is also explained in the Materials and Methods section (*"Mock group contained two or three animals, each of the RVFV-infected group contains six animals (MRU25010-30, MRU2687-3 and ZH548) and experiment was repeated twice except for ZH548.";* lines 247-250 in the revised manuscript), we did not amend the legend of Figure 3.

d. I was a little confused by how the significant differences were indicated. Looking at SC-10 (note, that really should be 10^1 to be consistent with the way you are indicating titer), it appears that the survival of mice inoculated SC with MRU2687-3 was not significantly different than those inoculated with ZH548 as indicated by brackets. However, the two survival rates are clearly significantly different.

We apologize for the confusion. We indicated in Figure 3 when there is no statistical difference ("ns") according to Gehan-Breslow-Wilcoxon test used in our study. When comparing survival rates of mice infected with either ZH548 or MRU2687-3 in SC-10^{1} condition, the p value is 0.0541. To note, we decided to use Gehan-Breslow-Wilcoxon test for statistical analysis (as indicated in materials and methods section, lines 255-257 in the revised manuscript) as this method gives more weight to deaths at early time points. This is particularly important here as the dynamics of mice death induced by MRU25010-30 strain is generally faster than that induced by the two other strains.

e. Here and throughout the manuscript, "p<0,05" should be "p<0.05" as in English usage, a

period, rather than a comma, is used to indicate a decimal. Please change all.

Thank you very much for spotting these typing mistakes. We corrected the manuscript accordingly.

6. Lines 392-394: Yes, the LD/50 was 9 days for both doses. However, the mortality rate was 67% for those inoculated with10^1 PFU as compared to 100% for those inoculated with 10^3 PFU, so there does appear to be a dose effect on mortality.

By comparing the dose effect on survival rates of ZH548-infected mice, the p value given by Gehan-Breslow-Wilcoxon test is 0.5055.

7. Lines 394-395: For both routes the mortality rate was 100% and the LD/50's were 8 versus 9 days, i.e., one mouse died one day earlier in one group. Is that really significant?

Indeed, the p value is 0.0411. For more clarity we changed $p<0.05$ to $p=0.0411$ at line 413 in the revised manuscript.

8. Line 404: Yes, you used RT-PCR to detect RVFV RNA, but were any of the samples actually checked to confirm that they contained infectious RVFV. Unfortunately, noninfectious pieces of RNA may persist in the blood producing a false positive if only RNA is sampled. I would be surprised if any of the mice were viremic on day 5 after infection despite the presence of RVFV RNA.
9. Lines 415-416: Again, checking for RNA does not check for virus or viremia. Did any of the mice contain virus in their blood when they died on or after day 5?

We agree with reviewer #2 that presence of RVFV RNA in the serum does not necessary correlate with presence of infectious viral particles, especially at later time pi. To note, it has been previously showed that Balb/c mice SC-infected with ZH501 strain still have viremia until D6 pi although the authors failed to detect infectious viral particles in the sera at D8 pi (Smith *et al.*, 2010). Our study was designed to follow level of RVFV RNA, anti-RVFV IgM and IgG through the time. For ethical reason, we did not collect more serum per animal at each time point (50 µl), so we were unable to titrate the amount of infectious RVFV in the sera.
To prevent any confusion or mislead readers, we changed "viremia" to "RNAemia" throughout the manuscript. Importantly, we added in the revised manuscript a set of data showing the viral infectious titres in liver and brain of mice that were positive for viral RNA by RT-qPCR in these organs (Figure 5), please see point 12 below.

10. Lines 417-423 (Figure 4): Again, why write "10PFU (10) or 103PFU (103) of…" instead of "10^1 or 10^3 PFU of…?"

We did the modifications.

11. Line 427: Did any of these six mice that failed to produce IgM have a viremia, i.e., were they infected with RVFV?

In SC-10^3 group, the mouse that failed to produce IgM did not show RNAemia, when one

mouse produced IgM without detectable RNAemia. For the five SC-infected mice with 10^1 PFU of MRU2687-3, we detected a transient RNAemia in the serum of one mouse at D3 pi but not in those of the other four mice. To note some of the mice that were infected with the same dilution of MRU2687-3 viral stock (i.e. same experiment) eventually showed RNAemia, seroconverted and have been euthanised as they were moribund. Additionally, we also detected viral RNA, as well as infectious viral particles (all but one mouse), in the brain of the euthanised mice. Altogether, we are confident that these mice were experimentally infected, but it did not lead to a productive RVFV infection in these animals.

12. Lines 434-437: Why weren't you able to test more mice? Based on Table 3, six mice infected with 10^3 PFU of MRU25010-30 SC died on day 4? Even more importantly, did you confirm that the RNA detected in that mouse actually represented infectious RVFV?

Reviewer #2 is right, as shown in Figure 3, six mice died at D4 pi. However, only one have been euthanised when the other five mice were found dead. As good experimental practice, we do not collect organs and sera from dead animals (but only from those that have been euthanized) since the quality of the samples may impaired subsequent analyses (due to autolysis of the liver for example). To note, the only mouse euthanised at D4 pi showed infectious viral particles both in the liver ($1.23x10^6$ PFU/g) and the brain ($2.22x10^3$ PFU/g). We modified Figure 5 to include the viral titres, expressed as PFU/g, in the liver and brain of mice that were positive for viral RNA by RT-qPCR. We also amended (i) the materials and methods section as follows: "*Viral RNA-positive brain and liver samples were also titrated by plaque assays, and viral titres were expressed as PFU/g*." (lines 254-255 in the revised manuscript); (ii) the results section as follows: *"Next, we tested the presence of infectious RVFV particles in the viral RNA-positive liver and brain samples by plaque assays. Overall, we observed similar patterns between viral infectious titres and RT-qPCR data (Figure 5)."* (lines 459-462 in the revised manuscript). Indeed, these data showed that (i) infectious RVFV particles are detectable at early time point post-infection (i.e., D3-6 pi) in the brain of one mouse infected by MRU25010-30 strain, independently of the dose or the route of inoculation; although these viral titres are lower than that measured in the liver and (ii) at later time post-infection (i.e. D7-10 and D11-15 pi), viral loads are higher in the brain than in the liver for all the three RVFV strains. Therefore, as requested by Reviewer #2, we confirmed that the mice infected with RVFV in our study sustained an active viral replication leading to the production of infectious viral particles.

13. Lines 452-359: If the purpose of this study was to compare the two strains of RVFV, why did you test viremias and the presence of virus in livers and brains on different days for the two strains? Again, the mere presence of viral RNA does not mean that any infectious virus was present.

The main explanation lies in the fact that the infection dynamics of the two strains are very different. In one hand, as discussed above, we were able to analyse only a small number of mice infected with MRU25010-30 at short times post-infection, due to the sudden death of the animals. Moreover, since RNAemia and levels of viral RNAs in liver and brain of mice infected with MRU25010-30 were relatively high at shorter time point pi, we aimed to refine viral replication kinetics and organ tropism at D1, D2 and D3 pi. On the other hand, given the

data already obtained with MRU2687-3 strain, we decided that it was not necessary to measure levels of viral RNAs in serum, brain and liver samples at D1 and D2 pi, but that it was more useful to investigate a later time point prior the median survival (i.e D6 pi). The same is true for ZH548 strain, but since this strain seems to be more virulent when IN inoculated (compared to SC infection), and comparable to MRU25010-30 strain in this condition, we decided to include an earlier time point (i.e. D2 pi) in our study. Altogether, we made sure to have a least one common time (D3 pi) between all the three RVFV strains to keep our comparative analysis. Concerning the presence of infectious particles please see previous comment.

14. Lines 515-516: Despite what was said here, time to death was also associated with dose and mice given the larger dose died sooner.

We agree with Reviewer #2 that this part of the discussion was not very clear. It is more relevant to indicate the effect of the dose on the time to death. Therefore, we modified the text as follows: *"Moreover, it has been previously shown that RVFV infectious dose is modulating the time of death (Graham et al., 2023; Kreher at al., 2014; Gowen et al., 2013)."* (lines 538-539 in the revised manuscript)

15. Lines 521-530: Interesting. Looking at Figure 6, how could there be virus in the brain of one of the SC inoculated mice on day 1 if there was no virus detected in the blood of that mouse? I would bet that there was an error in the laboratory.

We were also surprised by this result. Therefore, we (1) repeated the RNA quantification with a larger volume of serum and (2) re-extracted RNA from brain sample before to quantify RNA level by RT-qPCR. Our initial results were confirmed, therefore we validated it.

16. Lines 566-569: Was this a subpopulation within the MRU2687-3 strain that differed or was it the 5'UTR of segment M at position 10 of the MRU2687-3 strain that differed?

We clarified this point by modifying the sentence as follows: *"Interestingly, deep-sequencing analysis combined with RACE-PCR approach allowed us to unveil a subpopulation at nucleotide position 10, which can be either an uracil or a cytosine, in segment M (antigenome) of both MRU2687-3 and ZH548 strains."* (lines 589-592 in the revised manuscript)

17. References: These need to be formatted properly.
a. Only the first word and proper nouns in a reference title should be capitalized. See references 2, 13, 25 and many others.
b. Proper nouns need to be capitalized. See references 5, 8, 9, and many others.
c. For scientific names, the genus should be capitalized, the species not capitalized, and the species name should be in italics. See references 3 and 39. See also reference 20 where there are extra spaces before and after the scientific name.
d. Why is the name of the editor of the various PLoS journals included
e. Shouldn't PLOS ONE or PLoS ONE be PLoS One? See references 52, 54, and 79?

References have been formatted as indicated by Reviewer #2.

Minor Comments:

18. Line 34: "has been isolated in camel" should be "was isolated from a camel…" Similarly, "isolated in goat" should be "isolated from a goat…"

Thanks, we already modified this as suggested by Reviewer #1: *"The first strain, MRU25010-30, was isolated from a camel (2010) while the second, MRU2687-3, was isolated from a goat (2013)."* (lines 34-35 in the revised manuscript)

19. Lines 38, 39: All results presented in a paper should be in the past tense, so "analysis shows…" should be "analysis showed…" and "we show that…" should be "we showed that…" please check the remainder of the manuscript as there are numerous results and conclusions in the present tense.
20. Line 84: Shouldn't "an RVFV outbreak…" be "a RVFV outbreak…"
21. Line 116: What are "ORFs?" I know that you are referring to open reading frames, but if you are gong to establish "nts" then you should establish ORF for "open reading frames." As it is used again.
22. Line 148: The "2" in "CO2" should be a subscript.
23. Line 150: As the strain was only isolated one time, it should be "…strain was isolated…" Same comment for lines 152, 154, and 155.
24. Line 180: It is probably better to use "were extracted…" rather than "have been extracted… Again, throughout the manuscript, virtually all of the "have been" should be "were…"
25. Line 199: why use "75nt" instead of "75nts? Earlier, line 100, you established nts to mean nucleotides.
26. Line 215: Because "12" is being used to modify "well," it should be "12-well format…" or probably better to simply state, "12-well pates and…"
27. Line 218: Why capitalize "Plaque Forming Units?" It should be "plaque-forming units (PFU,…" Note the hyphen between plaque and forming.

We thank Reviewer #2 for these suggestions and we modified the manuscript accordingly.

28. Line 222: Again, why capitalize "Tissue Culture Infectious Dose?" These are not proper nouns. Also, the 50 in TCID50 should be a subscript.

We modified it throughout the manuscript as well as in Fig. 1

29. Line 231: As there was more than one dose, it should be "were either…"
30. Line 232: Numbers, not of measurement, less than 10 should be written out, so this should be "two or three animals…" and six animals…" See also line 244, 252, 366, 457, and many others.
31. Line 240: Despite the above comment, this should be 6 to 8 weeks as weeks are a unit of measurement. Please also see line 229 and others.
32. Line 270: Again, it is probably better to use "were obtained…"
33. Line 282: Should "99%" be "99.0%" as the others are given to a tenth of a percent?

We thank again Reviewer #2 for all these suggestions and we modified the manuscript accordingly.

34. Line 335: Why establish HepaRG here? It should have been established the first time it was use on line 137.

It was indeed established in the Materials and Methods section but, for the readers, we think it is important to specify again the origin of this cell line within Results section.

35. Line 349: As was used later in this same sentence, use "were infected" instead of "have been infected…"
36. Line 357: why establish "SD" if it is not used again?

We corrected the manuscript as proposed by Reviewer #2.

37. Line 365: results should always be in the past tense, so this should be, "we showed that…" See also line 368 where "reaches…" should be "reached…", line 470 where "we show…" should have been "we showed…" and several others. Please check the entire manuscript.

We carefully checked and modified the entire manuscript.

38. Line 377: Why establish IN and SC again here? They were established on line 230.

Again, we think it will help the readers if they did not carefully read the Materials and Methods section at the first place.

39. Line 381: Why is it "Six-eight weeks" here and "6-8 weeks…" in Figures 4 and 6?

We apologize for this inconsistency and we changed it as suggested by Reviewer #2.

40. Line 410: "figure 3" should be "Figure 3."
41. Lines 435, 460, and many others: It is more accurate to use, "was…" rather than "has been…"

We carefully checked and modified the entire manuscript.

2. Line 470: Why is there a space after the "7" in MRU2687 -3?
43. Line 536: Why reestablish "RdRp" when it was already established on line 100?
44. Line 509: Because there was more than one mouse, "than the one…" should be "than the ones…"

Thank you very much for spotting these errors. We corrected the manuscript accordingly.

Reviewer #3:

We thank reviewer #3 for her/his useful suggestions and we are grateful for the following supportive comment: "The experiment is appropriately conducted and the results are articulated very well with great clarity". However, Reviewer #3 stated: "because of only one sample being analysed, the conclusion that it is highly virulence needs additional support, for example from mutation analysis."

Specific comments

1- The attempt to perform intra-strain genetic diversity analysis through mutation profiling has been extremely shallow based only on observation where amino acid differences were simply listed rather than any serious attempt to infer functional information. A more indepth analysis of the non-synomous mutations should have generated some speculations for example those that may be associated with virulence or vice versa.

We observed that most of the intra-strain genetic diversity was located in the Gn gene although some diversity was also observed in the N, NSs, L and Gc genes as detailed in Table 1. We also identified an interesting mutation within the 5'UTR of segment M (antigenome), for both MRU2687-3 and ZH548 strains, leading *"to an additional initiation AUG codon located upstream of AUG1, which has already been described for MP-12 vaccine strain".* We discussed these observations at lines 583-593 in the revised manuscript. However, by comparing the data obtained for the three RVFV strains characterised in this study, and based on our current knowledge, we were unable to speculate further on the importance of a given non-synonymous mutation. Moreover, in line with Reviewer #3 comment, we discussed that point at lines 596-601 in the revised manuscript: *"Indeed, the impact of the intra- and inter-strain genetic diversity on the striking differences observed between the two field strains remains unknown. However, it will be now possible to develop specific reverse genetics systems, based on our sequencing data, to decipher the effect of quasi-species as well as amino acid substitutions on the RVFV-induced pathogenesis, replication and interaction with host immune response, such as IFN signalling pathways."* Indeed, the use of reverse genetics system will certainly be a valuable asset to link with certainty specific non-synonymous mutations and/or the presence of viral quasi-species with RVFV virulence in mice.

2- The objective behind the RACE-PCR analysis is not clear. Was this to validate the whole genome sequencing or what was the data meant to achieve beyond whole genome analysis?

It is common to have low coverage of the 5' and 3' UTRs with deep-sequencing approach. Therefore, we use RACE-PCR method to validate the whole genome sequencing data at the 5' and 3' UTR. Since, we observed no difference between RACE-PCR and deep-sequencing approaches, we changed *refined* with *confirmed* in the sentence lines 292-293 in the revised manuscript: *"The sequences of the 5' and 3' ends of each segment were confirmed by RACE-PCR analysis."*

3- It is not clear to me what the "high virulence" here means given that these are only two single isolates being compared. Was there any functional element detected from the genomic analysis that could be associated with virulence or vice versa ….and rule out the possibility that the difference observed is due to sample source.

This comment is in line with general comments of Reviewer #1. Therefore, we changed the title of the manuscript to *"Comparative study of two Rift Valley fever virus field strains*

originating from Mauritania.", to ensure that our title does not mislead anyone into over-interpreting the data. To note, we also tested in a preliminary study another RVFV strain isolated from a camel during the 2010 outbreak in Mauritania. Survival curves were comparable to the ones showed for MRU25010-30 strain. However, at that time, we decided to focus our study on only one strain isolated from a camel alongside with MRU2687-3 and ZH548 strains. Moreover, we added the following sentence in the discussion to consider the limitations of our study: *"To date, given the number of RVFV strains tested in this study and the fact that no viral determinant can yet be associated clearly with the virulence observed in mice, it is difficult to extrapolate our data to other RVFV strains or lineages. Indeed, the impact of the intra- and …."* (lines 594-596 in the revised manuscript).

Minor comments

It is not clear how biosafety considerations were adhered to given the pathogenic and fatal nature of RVFV.

We had the following sentence in "Ethical statement" within Materials and Methods section: *"All experiments with infectious RVFV have been conducted by trained staff in certified biosafety level 3 laboratories and by following strict biosafety protocols."* (lines 268-270 in the revised manuscript)

Line 30: Consider changing 'rainfalls' to 'rains'

We corrected the manuscript as suggested by Reviewer #3.

Line 54-56: change 'In this study, we fully sequenced two RVFV strains isolated in Mauritania during two distinct outbreaks (2010 and 2013) and show that they were genetically distant.' to 'In this study, we sequenced two RVFV strains isolated in Mauritania during two distinct outbreaks (2010 and 2013) and obtained full-length genomes which are genetically distant'

We already changed this sentence, according to Reviewer #1 and #2 suggestions, as follows: *"In this study, we fully sequenced the genomes of two RVFV strains isolated in Mauritania during two distinct outbreaks (2010 and 2013) and showed that they were genetically distant."* (lines 54-56 in the revised manuscript)

Line 81: Consider replacing the word 'highly' with 'first'

Thanks for the suggestion. We changed the text as suggested.

Line 116: Consider providing the full word for ORFs before abbreviating it

We now provide the full word "open reading frames".

Line 119: 'located in' to 'located on'

We thank reviewer #3 for spotting this mistake that has been corrected.

Line 124: Change ‘We fully sequenced these two viral strains,…’ to ‘We obtained full genome sequences of these two viral strains,…’

We already changed this sentence to : *“We fully sequenced the genomes of these two viral strains, …”*

Line 128: ‘open’ to ‘offers’

We did this change in the revised manuscript.

Line 148: subscript the 2 in CO2 symbol

Line 150 ‘located’ to ‘sampled’

Line 152: as suggested for line 150 above

We thank reviewer #3 for these suggestions and modified the manuscript accordingly.

Line 154: ‘ISRA-LNERV’ is not defined. Is this the name of the lab where culture was performed?

We now defined ISRA-LNERV in the revised manuscript. We are also grateful to Reviewer #3 for her/his comment as we realized that this paragraph was inaccurate. Indeed, MRU25010-30 strain was isolated at ISRA-LNERV and MRU2687-3 strain at CIRAD laboratory. We changed this paragraph as follow: *“The MRU25010-30 strain was isolated from the serum of a sick camel sampled in Lemsayddi, within the desertic Adrar region of Mauritania during the 2010 outbreak [19,42], at “Institut Sénégalais de Recherches Agricoles-Laboratoire National de l'Elevage et de Recherches Vétérinaires” (ISRA-LNERV, Senegal). The MRU2687-3 strain was isolated, at the “French Agricultural Research Centre for International Development” (CIRAD, France), from the placenta of an aborted goat sampled in the Brakna region of Mauritania along the Senegal river basin, during the outbreak of 2013 [22]. These two strains were passaged twice in VeroE6 before being used in this study.”* (lines 151-158 in the revised manuscript)

Line 177-178: Insert a reference

We specified the company that sequenced the amplicons, i.e. Eurofins Genomics, Germany

Line 185: Add a comma after preparation

We had the coma

Line 186-188: Consider rewriting the sentence to best capture the end prep and ligation procedure.

We apologize as we did not clearly specify that we were following the manufacturer's guidelines. We changed the sentence as follows: *"The cDNA was processed through the End Repair step and the subsequent library preparation protocol according to the manufacturer's guidelines until the Adapter Ligation step. Briefly, following bead-based clean-up, the cDNA underwent End Repair at 20°C for 30 minutes. This was followed by another bead-based clean-up and A-tailing at 30°C for 60 minutes. After a third bead-based clean-up, the DNA fragments were incubated with NEB's diluted adaptor (0.15 µM) to accommodate the low nucleic acid input at 20°C for 60 minutes, followed by treatment with USER enzyme at 37°C for 15 minutes."* (lines 189-196 in the revised manuscript). We hope that this part is now clearer.

Line 200-202: Could you briefly describe the steps in the two pipelines. How were the variants called using the Sam2Consensus program? What thresholds were used for example on the read depths? This can be best captured if the steps can be summarized in a few sentences.

We have made appropriate changes in the methodology to reflect the pipeline parameters used for consensus generation and variant calling: *''All the reads with a minimum Phred score of 25 were used in the consensus calling. The minimum read depth used for consensus generation is 20 reads. In the genome terminal sequences, consensus sequences were curated with the majority read consensus. The low-frequency variants from the SAM (sequence alignment map) file were calculated using the Sameer program (https://github.com/vbsreenu/Sameer). All the amino acid variants with a read depth greater than 20 reads and frequency more than 5% are reported in our study."* (lines 210-216 in the revised manuscript). To note, we deposited the code used for the variant calculation on GitHub (https://github.com/vbsreenu/Sameer) as well as a demo usage for users to utilize our package.

Line 204-205: The sequences for the MRU25010-30 and MRU2687-3 are still unavailable. Only the ZH548 sequences can be found on NCBI GenBank database. I suspect that they will be released after the publication or after the period indicated by the sequence submitters.

Indeed, the sequences of both MRU25010-30 and MRU2687-3 strains were due to be released on Nov 10, 2024. They are now available on NCBI GenBank database.

Line 207: How were the protein sequences obtained? Were the nucleotide sequences translated in ClustalW or MEGA x after the nucleotide alignment procedure?

Nucleotide sequences were translated by using MEGA X software after the nucleotide alignment procedure. Moreover, we checked protein sequences with alternative softwares such Translate lools available in Expasy website (https://web.expasy.org/translate/) and, as expected, no difference was observed between the different methods used. Alignments generated by ClustalW were mainly used to determine the percentage of identity between nucleotide or protein sequences as indicated lines 293-296 and 302-304 in the revised manuscript.

Line 210: Insert the reference 45 in after (GTR) in line 209

We modified the manuscript as suggested by Reviewer #3: *"The evolutionary history was inferred using the maximum likelihood (ML) method and general time reversible model (GTR) [45] implemented in the above-mentioned MEGA X software."* (lines 223-225 in the revised manuscript)

Line 231: Consider writing PFU in full (Plaque Forming Units) before abbreviation.

We already did this modification, please see point 27 of Reviewer #2.

Line 235: Write pi in full, post-infection

"pi" is already defined earlier in the manuscript. However, we are grateful for Reviewer #3 comment, as there were some redundancies in the manuscript that have been corrected.

Line 270: Omit the word 'The' instead begin by 'Whole genome ….

The manuscript has been corrected.

Line 277: Consider performing lineage analysis using the recently developed tool based on Grobbelaar nomenclature (A-O) here https://www.genomedetective.com/app/typingtool/rvfv/ using the M segment sequences and https://github.com/ajodeh-juma/rvfvtyping for all the segments (Juma et al., 2022)

We tested the tool suggested by Reviewer #3 (Summary of the results are presented below) with sequences deposited in Genbank: OR844391 (MRU25010-30 Seg-M), OR844388 (MRU2687-3 Seg-M) and OR805806 (ZH548 Seg-M). No assignment was obtained with segments L and S of the three strains. The tool was able to assign ZH548 segment M to lineage A (based on Grobbelaar nomenclature) but failed to assign a lineage to both MRU25010-30 and MRU2687-3 strains. Looking at the phylogenetic tree provided (see below), MRU2687-3 Seg-M belongs to lineage H whereas the one of MRU25010-30 Seg-M appears to be closer to Lineage N.

To note, we already performed phylogenetic analysis based on Grobbelaar classification before the submission of the original manuscript, and obtained similar results. However, "rvfvtyping genome detective" is a bioinformatics analysis pipeline for classification and phylogenetic lineage assignment of Rift Valley fever virus consensus genomes using the glycoprotein Gn/G2 gene encoded by Seg-M. In our case, since we generated the complete sequences for each segment of the three RVFV strains characterized in our study, we think that it is more relevant to use Bird and colleagues' classification, in order to capture the entire genetic diversity of each viral segment; even if less RVFV sequences are available to perform the analysis.

École Pratique des Hautes Études PSL

SUMMARY

Blast/aga assignment	Genotype assignment	Sequences count	Percentage	Legend
Phlebovirus riftense		6	66.7%	
	Could not assign	2	22.2%	
	Lineage A	1	11.1%	
Total		9	100%	

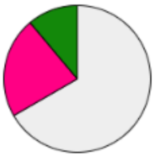

Name	Length	Blast	Lineage	Report	Genome
**OR844390.1**	1691	Phlebovirus riftense		Report	
**OR844391.1**	3885	Phlebovirus riftense	Could not assign	Report	
**OR844392.1**	6404	Phlebovirus riftense		Report	
**OR844387.1**	1689	Phlebovirus riftense		Report	
**OR844388.1**	3885	Phlebovirus riftense	Could not assign	Report	
**OR844389.1**	6404	Phlebovirus riftense		Report	
**OR805807.1**	1690	Phlebovirus riftense		Report	
**OR805806.1**	3885	Phlebovirus riftense	Lineage A	Report	
**OR805805.1**	6404	Phlebovirus riftense		Report	

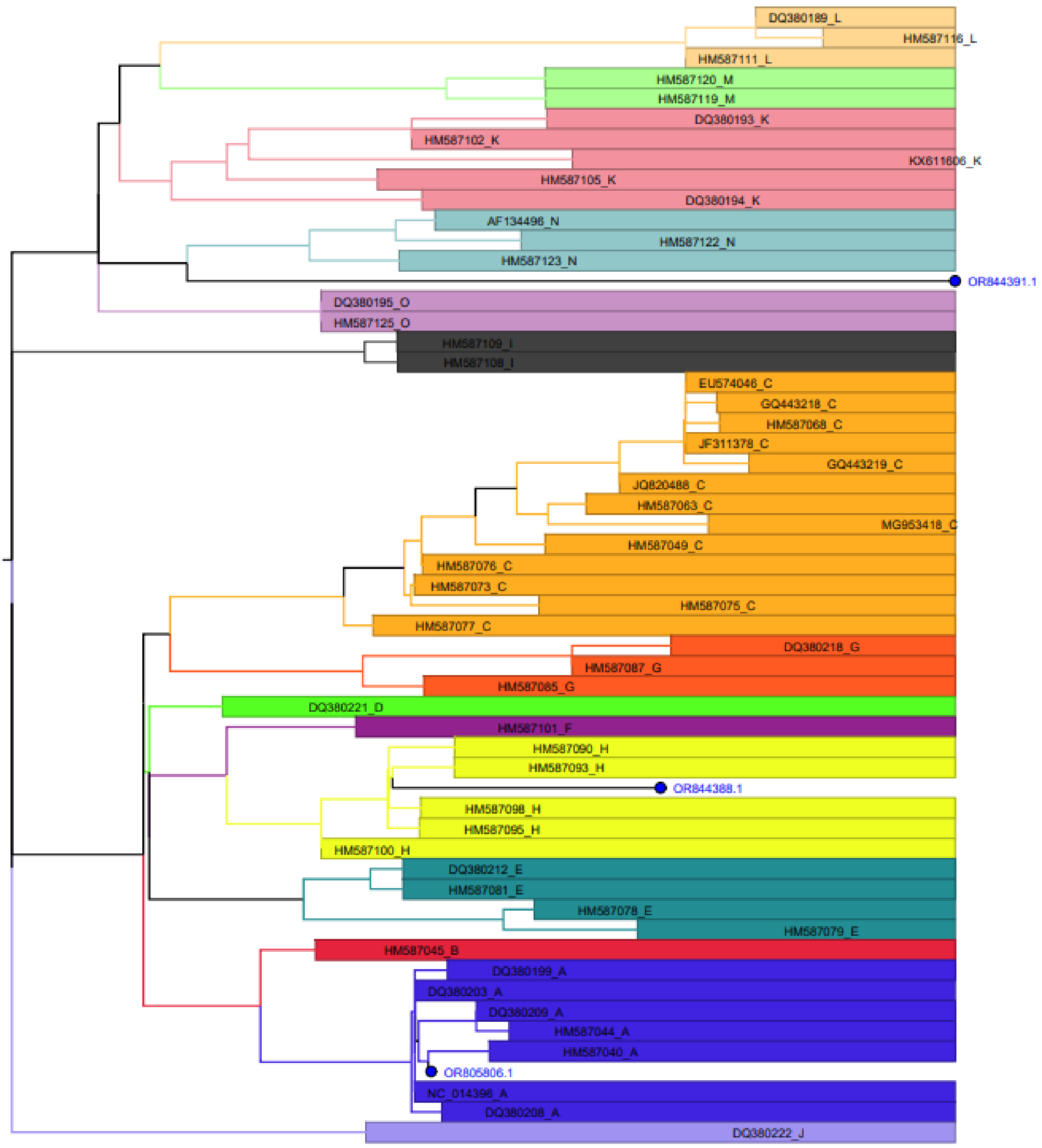

Line 295: The model used here is Jukes-Cantor while in the methods, specifically line 209, the authors specify GTR model. Could the authors clarify about this? In the sequence analysis as displayed on the figure, what strain was used as reference to identify the amino acid substitutions?

We sincerely apologize for this mistake, and we are grateful to Reviewer #3 to have spot it. The Materials and Methods section is correct, and we changed “Jukes Cantor model” to “GTR model” in the legend of Figure 1.

Figure 1 panel B is highlighting amino acid substitutions between the two field strains only. As

mentioned in the legend: *"Position and amino acid residues of MRU25010-30 (left) and MRU2687-3 (right) strains are indicated."* (lines 322-323 in the revised manuscript)

The authors can compare the amino acid substitutions not just between the two strains from Mauritania but also between the 'virulent' strain versus all the other sequences used in the study to have a global picture of the changes.

We already compared amino acid substitution between MRU25010-30 and ZH548 strains that is considered as a virulent strain in the literature (please see Table S2) and discussed this point (lines 554-582 in the revised manuscript). However, even if we agree with Reviewer #3 comment, we think that it would be too speculative, at this stage, to make such comparison.

Overall, the manuscript is well written. Further investigations especially looking at the amino acid residues substitutions may be relevant to firmly link the observed changes to the virulence or replicative properties of the virulent strain that is reported here.

We agree again with Reviewer #3 comment and this is why we proposed such investigations within the discussion by using, for example, reverse genetics system (lines 596-601 in the revised manuscript).

Finally, I would like to thank you and the reviewers again for the useful comments and suggestions. We trust we have addressed the comments/suggestions of the referees appropriately and we believe that the manuscript is much improved and now ready for publication in PLOS Neglected Tropical Diseases.

Yours sincerely

Dr Maxime Ratinier, Senior Lecturer
BUNYA Team Leader, UMR754 IVPC (Viral infections and Comparative pathology)
UCBL1 INRAE EPHE
