# Supplementary material for: Comparative study of two Rift Valley fever virus field strains originating from Mauritania
Source: PLoS Negl Trop Dis. 2024 Dec 9;18(12):e0012728. doi: 10.1371/journal.pntd.0012728 (PMC11658707; doi:10.1371/journal.pntd.0012728)
Supplement: Attachment — Submitted filename: Rebuttal_Letter_Chabert_et_al_2024_R2.pdf [file pntd.0012728.s013.pdf]

Brian Byrd, Guest Editor

David Safronetz, Section Editor

PLOS Neglected Tropical Diseases

19 November 2024

Dear Editors

RE: Comparative study of two Rift Valley fever virus field strains originating from Mauritania.

We would like to thank you and the reviewers again for the time spent on our manuscript and for the helpful recommendations and suggestions.

Below, we provide a "point-by-point" reply. In order to facilitate the editorial process, we inserted our replies directly underneath the relevant text of the reviewers (in red), which we cut and pasted from the Editors' letter. In addition, as recommended, we provided two versions of the revised manuscript: one with track changes denoting where the text has been changed and a clean version uploaded as manuscript file.

Reviewer #1:

"This manuscript contains information that is of interest to the readership of this journal. The revised version is acceptable for publication."

We thank Reviewer #1 for his supportive comments.

Reviewer #2:

General Comment: The revised manuscript is much improved. Thanks for including the data on the presence of actual infectious virus. Below, in addition to a few comments that need to be addressed, are some suggested word changes to improve the "English" in the manuscript.

We would like to thank again Reviewer #2 for her/his useful comments.

Specific Comments:

1. Lines 443-444: Given that five of the six mice that failed to produce IgM also failed to produce detectable RNA in their sera, isn't it very likely that those mice were never infected with the MRU2687-3 strain of RVFV despite being inoculated with that virus? Did you detect virus or RNA from any of the organs? If not, I would assume that it is more likely that those mice were never infected with the MRU2687-3 strain of RVFV, even if they had been inoculated with that strain.

In SC-10^1 group, one of the five mice that failed to produce IgM showed a transient RNAemia at D3 pi, whereas the other four mice failed to produce detectable RNAemia in their sera. We agree with Reviewer 2, as stated in the previous rebuttal letter, that despite being SC-inoculated with MRU2687-3 strain, those mice had never been infected with that strain in this condition. To clarify this point, we added the following sentence (lines 443-447): "*In SC-10^1 group, one of the five mice that failed to produce anti-RVFV IgM showed a transient RNAemia at D3 pi, whereas the other four mice failed to*

produce detectable RNAemia in their sera, suggesting that MRU2687-3 inoculation did not lead to a productive RVFV infection in these animals."

2. Line 457: Were you testing for MRU25010-30 virus or MRU25010-30 RNA. I would indicate that it was RNA.

We indicated RNA as suggested by Reviewer #2.

3. Line 509: Unfortunately, Figure 6 is a bit misleading. Looking at the figure, it appears that MRU25010-30 replicates faster in the serum and liver than in mice inoculated SC with the MRU2687-3 strain. However, this difference is due to no mice inoculated with MRU2687-3 being tested before day 3. If the goal of that study was to compare the two strains, they should have been tested at the same time periods.

The aim was not so much to compare these two strains (although one time point of analysis is the same for both), but rather to gain more insight into the dynamics of infection of each strain. We agree that this figure can be visually confusing when read quickly. Therefore, in order to avoid misleading the reader, we modified Figure 6 as follows, where the data obtained for each strain are separated.

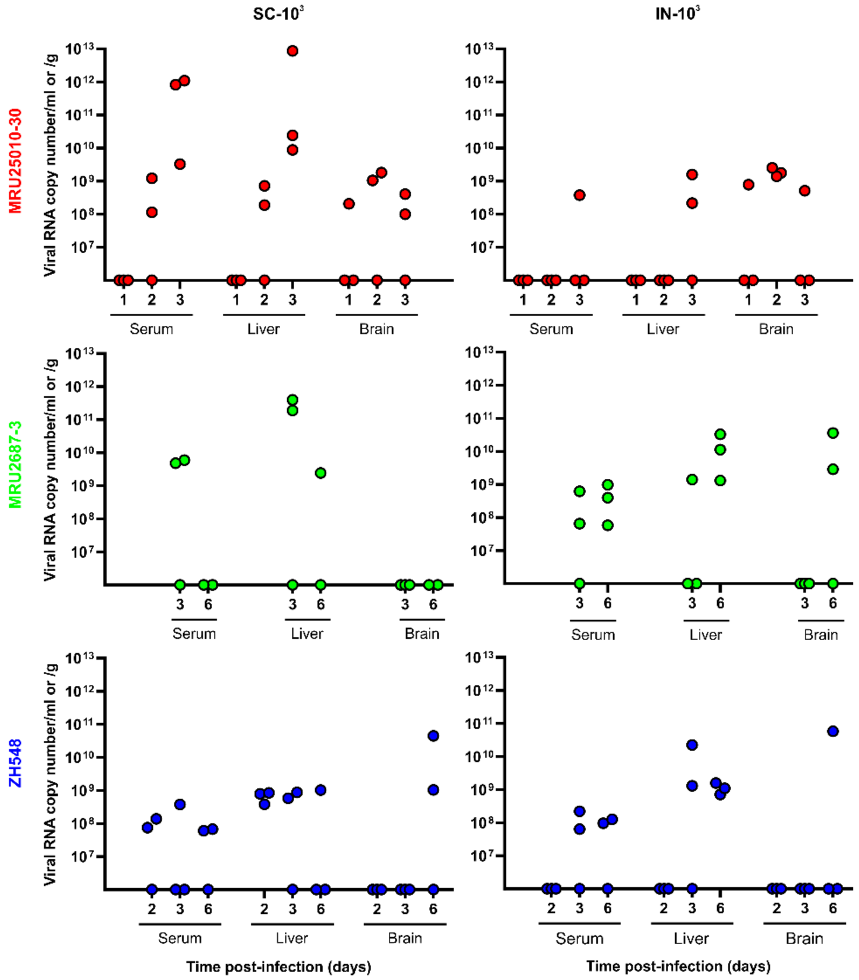

4. Line 529: What are "WF rats?"

WF rats is now written in full: « Wistar-Furth rats »

5. Lines 351-353: If the mouse inoculated IN with MRU25010-30 had no virus in the serum on day 1 and yet had a high brain titer, how did the virus get to the CNS if not directly from the olfactory tract?

We thank Reviewer #2 for highlighting this part of the discussion. We agree that this sounds too conclusive. Notably, we also observed the same pattern (i.e., detection of MRU25010-30 RNA in the brain but not in the serum at D1 pi) but in one SC-inoculated mouse. It is therefore likely that mechanism(s) other than direct passage of the virus through the olfactory neurons after IN inoculation are involved to explain this result and further studies are needed to understand the dissemination of this strain to the CNS depending on the route of infection. We modified the discussion as follows: " *of MRU25010-30 strain in vitro in HepaRG cells. Since, we also detected MRU25010-30 in the brain of infected mice as soon as 1 day pi, we cannot rule out that viral replication in this organ contributes to the pathogenesis induced by this strain. It is also known that RVFV targets the olfactory neurons lining the nasal tract, and BALB/c mice or Lewis rats exposed to aerosols or intranasally infected with RVFV have been shown to develop a much earlier and severe neuropathology than subcutaneously injected animals [17,58,59]. In our study, we did not observe difference in term of virulence between IN and SC conditions for MRU25010-30 and MRU2687-3 strains. Therefore, further studies are needed to investigate the dissemination and viral pathogenesis in the central nervous system depending on the route of inoculation and the field strain used." (Lines 547-556)*

Minor comments:

6. Line 36: Why stablish "RACE-PCR" if it is not used again in the abstract? You should not establish an abbreviation if it is not used again. See also line 154 for "ISRA-LNERV" and line 155 for "CIRAD."

7. Line 53-54: When writing a list, i.e., "comment a, comment b, and comment c" always include a "," after the item just before the "and" so "replicative capacities and transmission…" should be "replicative capacities, and transmission…" See also line 69, 71, 88, 107, 123, 135, 141, 167, 275, 291, 304, and others.

8. Line 91: Shouldn't "under evaluated" be "under reported?"

9. Line 162: "Two or three-days…" should be "Two or 3-days…" as numerals are used for all units of measurement except at the beginning of a sentence. Note, on line 244, you wrote "6 to 8 weeks old…" at the beginning of the sentence. You need to be consistent. I would have used "Six- to 8-week-old female…"

10. Line 246: I thought that you had corrected this. It should be "either 10^1 or 10^3 PFU." See also lines 400 and 436 where "10PFU (10^1) or 10^3PFU (10^3)"…" should be "10^1 or 10^3 PFU…"

11. Line 258: "1000 PFU…" should be "10^3 PFU…" Remember, I cannot use a superscript in this program.

12. Line 273: "3 times…" should be "three times…"

13. Line 364: Is it "enhanced viral ability to replicate" or "enhanced ability to replicate in viral cells?"

14. Line 384: "fifteen times more…" should be "15 times more…"

15. Line 416: It should be "with MRU2687-3 or ZH548,…" because you did not inoculate and mice with both MRU2687-3 and ZH548.

16. Line 444: Shouldn't "MRU2687…" be "MRU2687-3…?"

17. Line 454: Should "≈10^10-10^12 versus ≈10^8-10^9 copies…" be "≈10^10-12 versus 10^8-9 copies…?" See also line 479.

18. Lines 464-465: It might be better to state, "In contrast, the pathogenicity…"

19. Line 478: “two out three mice…” should be “two out of three mice…”

20. Line 507: Yes, it may enter “into” the brain later, but it is detected “in the brain…”

21. Line 526: Is “regrouping” the right word? Would it be better to write, “lineage A, consisting mainly of Egyptian and Namibian isolates, including the ZH548 strain…?”

22. Line 529: Why establish “LD50” if you do not use it again?

23. Line 785: Only the first letter of the first word (and proper nouns) should be capitalized. See also line 803 for Bovine Viral diarrhea virus, where neither bovine nor viral is a proper noun.

24. Line 899: “Rift Valley” should be capitalized.

In accordance with the above suggestions from reviewer #2, we have amended the manuscript.

Reviewer #3:

The authors have revised the manuscript well and addressed most of the concerns. The only part that has not been fully addressed is why the lineage assignments could not be replicated with other tools. This however doesn’t prevent the current publication but require a followup by the RVF experts.

We thank Reviewer #3 for his comments. It is known that RVFV lineage assignments depend on the phylogenetic analysis performed. This choice is mainly determined by the availability of sequences either full-length or partial, as well as the viral segment. We agree that the RVFV community will benefit from follow-up and further harmonized phylogenetic studies.

We trust we have addressed the comments/suggestions of the referees appropriately and we believe that the manuscript is now ready for publication in PLOS Neglected Tropical Diseases.

Yours sincerely

Dr Maxime Ratinier, Senior Lecturer

BUNYA Team Leader, UMR754 IVPC (Viral infections and Comparative pathology)

UCBL1 INRAE EPHE
