# Supplementary material for: Comparative study of two Rift Valley fever virus field strains originating from Mauritania
Source: PLoS Negl Trop Dis. 2024 Dec 9;18(12):e0012728. doi: 10.1371/journal.pntd.0012728 (PMC11658707; doi:10.1371/journal.pntd.0012728)
Supplement: Attachment — Submitted filename: manuscript_reviewer_comments.docx [file pntd.0012728.s011.docx]

The Study by Chabert et al has compared virulence of two RVFV isolates, one from a camel and the other from goat. The two samples have been cultured in vitro and used to infect mice for in vivo infections studies. The samples were also sequenced and whole genome data used to determine lineage classification.

The experiment is appropriately conducted and the results are articulated very well with great clarity.

**I have a few concerns:**

1. The attempt to perform intra-strain genetic diversity analysis through mutation profiling has been extremely shallow based only on observation where amino acid differences were simply listed rather than any serious attempt to infer functional information. A more indepth analysis of the non-synomous mutations should have generated some speculations for example those that may be associated with virulence or vice versa.
2. The objective behind the RACE-PCR analysis is not clear. Was this to validate the whole genome sequencing or what was the data meant to achieve beyond whole genome analysis?
3. It is not clear to me what the “high virulence” here means given that these are only two single isolates being compared. Was there any functional element detected from the genomic analysis that could be associated with virulence or vice versa ….and rule out the possibility that the difference observed is due to sample source.

**Minor comments:**

It is not clear how biosafety considerations were adhered to given the pathogenic and fatal nature of RVFV.

Line 30: Consider changing ‘rainfalls’ to ‘rains’

Line 54-56: change ‘In this study, we fully sequenced two RVFV strains isolated in Mauritania during two distinct outbreaks (2010 and 2013) and show that they were genetically distant.’ to ‘In this study, we sequenced two RVFV strains isolated in Mauritania during two distinct outbreaks (2010 and 2013) and obtained full-length genomes which are genetically distant’

Line 81: Consider replacing the word ‘highly’ with ‘first’

Line 116: Consider providing the full word for ORFs before abbreviating it

Line 119: ‘located in’ to ‘located on’

Line 124: Change ‘We fully sequenced these two viral strains,…’ to ‘We obtained full genome sequences of these two viral strains,…’

Line 128: ‘open’ to ‘offers’

Line 148: subscript the 2 in CO2 symbol

Line 150 ‘located’ to ‘sampled’

Line 152: as suggested for line 150 above

Line 154: ‘ISRA-LNERV’ is not defined. Is this the name of the lab where culture was performed?

Line 177-178: Insert a reference

Line 185: Add a comma after preparation

Line 186-188: Consider rewriting the sentence to best capture the end prep and ligation procedure.

Line 200-202: Could you briefly describe the steps in the two pipelines. How were the variants called using the Sam2Consensus program? What thresholds were used for example on the read depths? This can be best captured if the steps can be summarized in a few sentences.

Line 204-205: The sequences for the MRU25010-30 and MRU2687-3 are still unavailable. Only the ZH548 sequences can be found on NCBI GenBank database. I suspect that they will be released after the publication or after the period indicated by the sequence submitters.

Line 207: How were the protein sequences obtained? Were the nucleotide sequences translated in ClustalW or MEGA x after the nucleotide alignment procedure?

Line 210: Insert the reference 45 in after (GTR) in line 209

Line 231: Consider writing PFU in full (Plaque Forming Units) before abbreviation.

Line 235: Write pi in full, post-infection

Line 270: Omit the word ‘The’ instead begin by ‘Whole genome ….

Line 277: Consider performing lineage analysis using the recently developed tool based on Grobbelaar nomenclature (A-O) here <https://www.genomedetective.com/app/typingtool/rvfv/>

Using the M segment sequences and <https://github.com/ajodeh-juma/rvfvtyping> for all the segments (Juma et al., 2022)

Line 295: The model used here is Jukes-Cantor while in the methods, specifically line 209, the authors specify GTR model. Could the authors clarify about this? In the sequence analysis as displayed on the figure, what strain was used as reference to identify the amino acid substitutions?

The authors can compare the amino acid substitutions not just between the two strains from Mauritania but also between the ‘virulent’ strain versus all the other sequences used in the study to have a global picture of the changes.

Overall, the manuscript is well written. Further investigations especially looking at the amino acid residues substitutions may be relevant to firmly link the observed changes to the virulence or replicative properties of the virulent strain that is reported here.
