# Supplementary material for: Comparative study of two Rift Valley fever virus field strains originating from Mauritania
Source: PLoS Negl Trop Dis. 2024 Dec 9;18(12):e0012728. doi: 10.1371/journal.pntd.0012728 (PMC11658707; doi:10.1371/journal.pntd.0012728)
Supplement: Attachment — Submitted filename: Reviewer comments on PNTD-D-24-00500.pdf [file pntd.0012728.s010.pdf]

Reviewer comments on PNTD-D-24-00500

Comparative study of two Rift Valley fever virus field strains circulating in Mauritania in 2010 and 2013 reveals the high virulence of the MRU25010-30 strain isolated from camel by Mehdi Chabert *et a*l

General

The manuscript reports on the work done in comparing different strains of two Rift Valley fever viruses isolated at two different locations in Mauritania during two different outbreaks of Rift Valley fever

The manuscript is well-written; however, it could be improved to make it clearer and more accurate. For example, the use of the words virulence (lines 2 and 53) and pathogenicity (line 79 & so on) need to be context sensitive, such that the aspects relating to the virus and those relating to the host can be clearly understood.

The authors should bear in mind also that recent field isolates of pathogens tend to be more pathogenic. This decreases with passages and/or propagation *in vitro* or *in vivo*. Although the factors which underlie this are not understood, some could confound observations such as the ones made in this study.

Specific

**Line**	**Comment**
34-35	isolated from, not in
54 and 124:	it is the genomes of the viruses, not the viruses, that were sequenced
83	outbreak refers to disease, in this case Rift Valley fever, not RVFV
95	change “is” to “has been”.
124	insert the genomes of between the words sequenced and these
321	acids should be acid
433	replace have with had
435 & 472	replace has been with was
523	replace participates with contributes, etc

Materials and methods

These have been described in sufficient detail, making it easy to follow the results obtained, which are in turn well presented, both in figures and words.
There are a few grammatical inaccuracies, resulting from idiosyncrasies of the English language, but these can be easily rectified at the editing stage:

Results

These are well presented and are confined the data generated in the work.
The investigations were done both *in vivo* and *in vitro*, both producing complementary results.

Discussion and Conclusion

These are supported by the data obtained

Recommendation

Accept after minor editorial revision, with no need for another review.
